# Relationship between amino acid composition and gene expression in the mouse genome

**DOI:** 10.1186/1756-0500-4-20

**Published:** 2011-01-27

**Authors:** Kazuharu Misawa, Reiko F Kikuno

**Affiliations:** 1 Research Program for Computational Science, Research and Development Group for Next-Generation Integrated Living Matter Simulation, Fusion of Data and Analysis Research and Development Team, RIKEN, 4-6-1 Shirokane-dai, Minato-ku, Tokyo 108-8639, Japan; 2 Chiba Industry Advancement Center, 2-6 Nakase, Mihama-ku, Chiba 261-7126, Japan; 3 Kazusa DNA Research Institute, 2-6-7 Kazusa-Kamatari, Kisarazu, Chiba 292-0818, Japan

## Abstract

**Background:**

Codon bias is a phenomenon that refers to the differences in the frequencies of synonymous codons among different genes. In many organisms, natural selection is considered to be a cause of codon bias because codon usage in highly expressed genes is biased toward optimal codons. Methods have previously been developed to predict the expression level of genes from their nucleotide sequences, which is based on the observation that synonymous codon usage shows an overall bias toward a few codons called major codons. However, the relationship between codon bias and gene expression level, as proposed by the translation-selection model, is less evident in mammals.

**Findings:**

We investigated the correlations between the expression levels of 1,182 mouse genes and amino acid composition, as well as between gene expression and codon preference. We found that a weak but significant correlation exists between gene expression levels and amino acid composition in mouse. In total, less than 10% of variation of expression levels is explained by amino acid components. We found the effect of codon preference on gene expression was weaker than the effect of amino acid composition, because no significant correlations were observed with respect to codon preference.

**Conclusion:**

These results suggest that it is difficult to predict expression level from amino acid components or from codon bias in mouse.

## Background

Codon bias is a phenomenon that refers to the differences in the frequencies of occurrence of synonymous codons among different genes [1]. In the translation-selection model, natural selection is considered to be a cause of codon bias, because codon usage in highly expressed genes is biased toward "optimal" codons, i.e., codons corresponding to more abundant tRNAs in many organisms [2-6]. Methods have previously been developed to predict the expression level of genes from their nucleotide sequences, which is based on the observation that synonymous codon usage shows an overall bias toward a few codons called major codons [2-12].

Previous studies have provided clear evidence that the translation-selection model applies to some prokaryotes, such as *Escherichia coli *[6,13], but not to all bacteria [4]. Additionally, some evidence exists that suggests this model is also applicable to various eukaryotes, including *Saccharomyces cerevisiae *[14-17], *Caenorhabditis elegans *[18,19], and the fruit fly [16,20], and even to the vertebrate *Xenopus laevis *[21]. However, the relationship between codon bias and expression level as proposed by the translation-selection model is less evident in mammals [22-30]. Urrutia and Hurst [23] found a weak correlation between gene expression levels and codon bias in human, but failed to find a relationship between this correlation and tRNA-gene copy numbers.

Amino acid content is also known to be dependent on gene expression level in some bacteria [31,32], as well as in budding yeast [8]. To determine why the relationship between codon bias and gene expression level, as proposed by the translation-selection model, is less evident in mammals, we investigated the correlations between the expression levels of genes and both the amino acid contents of genes and codon preference, in mouse. Subsequently, we compared the effect of gene expression on codon preference to the effect of gene expression on amino acid composition. We used the expression data of mouse genes contained in the InGap database [33].

## Materials and methods

### Mouse Genes

We obtained cDNA sequences of genes of *Mus musculus *from the ROUGE (http://www.kazusa.or.jp/rouge/index.html) database [34]. In total, 449,444 codons from 1,182 genes were used. Mouse expression data were retrieved from the InGap database using cDNA microarray [33].

### Amino Acid Contents and Codon Preference

We calculated the proportion of the amino acid contents of all genes. In order to examine the translation-selection model, we classified amino acids into two classes, i.e., C- and T-adapted, on the basis of tRNA-gene copy numbers in the mouse genome, since tRNA-gene copy numbers can be considered as a rough estimate of tRNA abundance [26]. If natural selection is a cause of codon bias, codon usage in C-adapted amino acids of highly expressed genes will be biased toward C-ending codons and vice versa. First, we defined C-ending and T-ending codons; for instance, AGC is a C-ending codon and AGT is a T-ending codon. However, both encode the Ser residue. In the mouse genome, when the number of tRNAs complementary to C-ending codons for an amino acid is larger than the number of tRNAs that are complementary to T-ending codons, the amino acid is defined as a C-adapted amino acid. If the opposite is true, the amino acid is instead classified as a T-adapted amino acid. Furthermore, an amino acid is classified as T-adapted when the number of tRNAs that are complementary to C-ending codons is the same as the number of tRNAs that are complementary to T-ending codons. We obtained the number of tRNAs in the mouse genome from the GtRNAdb database [35]. Ser, Leu, Pro, Arg, Ile, Thr, Val, and Ala are T-adapted amino acids, whereas Phe, Tyr, Cys, His, Asn, Ser, Asp, and Gly are C-adapted amino acids. Of note, Ser is encoded by TCT, TCC, TCA, TCG, AGC, and AGT. The number of tRNAs that are complementary to TCT is larger than the number that are complementary to TCC, whereas the number of tRNAs that are complementary to AGT is smaller than the number that are complementary to AGC. We considered the two types of codons that specifically encoded Ser. We compared the expression levels of genes to the nucleotide composition at the 3rd position of the codons. We conducted this comparison for all amino acids, including the for T-adapted, and C-adapted amino acids

Because of CpG hypermutability, the mutation rates of codons are affected by the 3' adjacent codon [36,37]. Thus, the frequency of codon occurrence is dependent on the adjacent amino acid [36,38,39]. We analyzed the effect of adjacent nucleotides on amino acid composition. Specifically, we calculated the correlation between the proportion of the first and third nucleotides of the 3' adjacent codon in genes and the expression levels of those genes.

### Analysis

The Pearson product-moment correlation coefficients were calculated using *R *software [40]. Because the probability density functions of the amino acid contents, codon preference, and expression levels are not known, we used a Kendall test, which is a nonparametric correlation test. Some of highly expressed genes might have specific sequences and functions. Thus, we eliminated the outliers from the data; we defined outliers as both the 5% of genes with the highest expression levels and the 5% of genes with the lowest expression levels. We also conducted multiple-regression analysis.

## Results

### Correlation between amino acid contents and gene expression level

**Figure 1:**
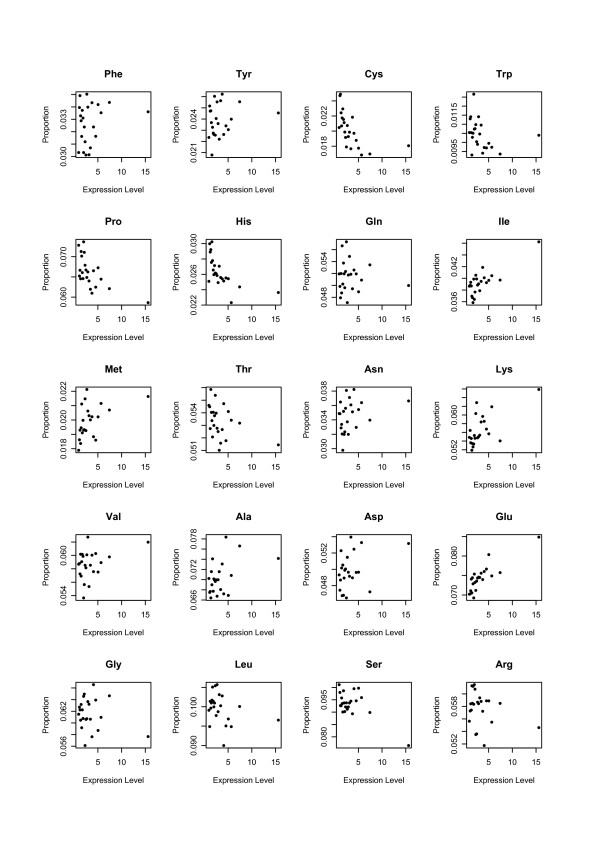
**Correlation between amino acid composition and gene expression level**. For preparation of this plot, we sorted the genes by their expression levels. The genes were sorted into bins of 50. Subsequently, the 50 genes were concatenated as a single large gene for analysis. Thereafter, the amino acid contents of the proteins were calculated. Each point on the plot represents a bin.

Figure 1 shows a scatter plot of amino acid contents and gene expression levels from the analysis of the mouse genome. Genes were sorted into bins of 50 genes by their expression level when this scatter plot was prepared. Subsequently, the 50 genes were concatenated as a single large gene, and the amino acid contents of the proteins were calculated. Each point on the plot represents a bin. For correlation analyses, we did not use these bins, but instead used each gene as a single entity. Figure 1 shows that the bin with highest expression level was exceptional. We also examined the sequence lengths, GC contents, and gene functions of highly expressed genes by using the ROUGE database [34], but we did not find any specific features.

Table 1 shows the correlation between amino acid composition and the gene expression level. The italicized numbers are the values that were calculated after eliminating the outliers. After the outliers were eliminated, the correlation test was performed. The contents of both Cys and His showed significant negative correlations with the expression level, whereas the content of Ile showed a significant positive correlation with the expression level. Multiple regression analysis showed that the multiple R^2 ^is 0.0797 and the adjusted R^2 ^is 0.06465 when all of amino acid components were used as predictors. After the outliers were eliminated, the multiple R^2 ^is 0.07193 and the adjusted R^2 ^is 0.0550 when all of amino acid components were used as predictors. These values of R^2 ^indicate that more than 90% of variation of expression levels cannot be explained by amino acid components.

**Table 1:** Correlation between amino acid abundance and gene expression level

	Overall			After Elimination of Outliers		
	
Amino Acid	Correlation coefficient	P-value		Correlation coefficient	P-value	
Phe	0.091	0.002	*	0.067	0.030	
Leu	-0.060	0.040		-0.047	0.124	
Ser	-0.123	0.000	**	-0.055	0.071	
Tyr	0.046	0.114		0.059	0.055	
Cys	-0.090	0.002	*	-0.118	0.000	**
Trp	-0.052	0.074		-0.031	0.319	
Pro	-0.080	0.006	*	-0.062	0.045	
His	-0.125	0.000	**	-0.169	0.000	**
Gln	-0.053	0.067		-0.010	0.757	
Arg	-0.061	0.036		-0.033	0.281	
Ile	0.135	0.000	**	0.084	0.006	*
Met	0.060	0.040		0.054	0.081	
Thr	-0.040	0.174		-0.045	0.141	
Asn	0.065	0.025		0.030	0.328	
Lys	0.091	0.002	*	0.031	0.308	
Val	0.091	0.002	*	0.069	0.024	
Ala	0.037	0.199		0.072	0.018	
Asp	0.101	0.000	**	0.054	0.079	
Glu	0.084	0.004	*	0.066	0.032	
Gly	0.004	0.884		0.018	0.567	

* Significant at 1% level

** Significant at 0.1% level

### Codon preference and gene expression level

Table 2 shows the correlation between gene expression levels and codon preference among mouse genes. This data revealed no significant correlations between codon preference and gene expression level. Thus, the effect of codon preference on gene expression was weaker than the effect of amino acid composition.

**Table 2:** Correlation between the nucleotide composition at the 3^rd ^position of codons and gene expression level

3' adjacent nucleotide	Overall	After elimination of outliers
All amino acids		
T	-0.063	-0.064
C	-0.061	-0.054
A	-0.068	-0.048
G	0.020	0.019
T-adapted amino acids		
T	-0.069	-0.068
C	-0.051	-0.033
A	-0.065	-0.050
G	0.012	0.000
C-adapted amino acids		
T	-0.049	-0.061
C	-0.061	-0.061
A	-0.054	-0.032
G	0.023	0.034

Table 3 shows the observed number of combinations of nucleotides at the third position and their 3' adjacent nucleotides. From this table, we can see the number of Cs at the third position of codons is significantly smaller than that of Ts when the 3' adjacent nucleotide is G (p < 0.1%, chi-square test). This finding indicates that codon preference in the mouse genome is affected by CpG hypermutability.

**Table 3:** Observed number of combinations of nucleotide at the third position and their 3' adjacent nucleotide

		The 3' adjacent nucleotide					
		
Codon type	Third nucleotide	T	C	A	G		H
All	T	45922	71142	48193	135728		165257
	C	88111	113015	141307	45671	**	342433
T-adapted	T	24963	42070	22892	64855		89925
	C	44589	54213	72472	21423	**	171274
C-adapted	T	20959	29072	25301	70873		75332
	C	43522	58802	68835	24248	**	171159

H stands for A, C, or T

** Significantly lower than the number of Cs when the 3' adjacent nucleotide is H (p < 0.1%) after Bonferroni correction when a chi-square test was used.

## Discussion

### Variation of gene expression level

There is a large variation among gene expression level [33]. More than 90% of variation of expression levels cannot be explained by amino acid components. Gene expression levels are known to be affected by many factors, such as 3'UTR lengths [41]. Further study must be necessary.

### Amino acid contents and gene expression level

To our knowledge, this is the first study that showed amino acid composition depends on the gene expression level in mouse. Previous study has shown that, in the case of budding yeast, some residues showed a positive correlation, and most of these residues were small [8]. Furthermore, Akashi and Gojobori [31] showed an increase in the abundance of less energetically costly amino acids in highly expressed proteins. This study also suggested that natural selection for energetic efficiency appears to constrain the primary structures of the proteins of *Bacillus subtilis *and *E. coli *[31]. Amino acid mutations that do not cause changes in protein functions may result in subtle, but evolutionarily important, fitness consequences through their effects on translation and metabolism.

We compared the estimates of the cost of amino acid synthesis from the above mentioned study [31] to the correlation coefficients presented in Table 1 (data not shown); however, we determined that the correlation was insignificant. It may be difficult to estimate the accurate metabolic cost of each amino acid, because the mouse obtains amino acids from food. Furthermore, the cost may depend on the environment. In the case of mouse, 10 amino acids, namely, Arg, His, Ile, Leu, Lys, Met, Phe, Thr, Try, and Val, are essential for natural growth [42]. Thus, sparing the incorporation of His and Ile in highly expressed proteins may be advantageous to the mouse. However, Cys is not an essential amino acid, and is negatively correlated with gene expression. Of note, both amino acid composition and gene expression level may be influenced by protein functions. Furthermore, adaptive changes in protein sequences may overcome the increases in the metabolic cost, and the amino acid sequences may not be optimized for metabolic cost. Further study is necessary to elucidate these issues. Our results show that the coefficient of determination is very small so that it would be hard to predict expression level from amino acid contents in mammals.

### Codon preference and gene expression level

We determined that the effect of codon preference on gene expression was weaker than the effect of amino acid composition, because no significant correlations were observed with respect to codon preference. This result is consistent with the relationship between codon bias and expression level, as proposed by the translation-selection model, is less evident in mammals [22-30]. In mammals, it would also be hard to predict expression level from codon bias.

### CpG hypermutability

Hypermutability of CpG dinucleotides [43] is one of major causes of codon substitution in mammalian genes [44-48]. CpG dinucleotides are often methylated at sites of cytosine (C); subsequently, the methylated C spontaneously deaminates to thymine (T) with a higher frequency than that of other types of point mutations [49]. It has previously been estimated that approximately 14% of codon substitutions are caused by hypermutations at CpG sites [36]. Furthermore, CpG hypermutation has been shown to affect the rate of amino acid substitution [39].

Table 2 shows that gene expression levels do not significantly affect codon preference in mouse. Furthermore, Table 3 indicates that the effect of codon preference is weaker than that of CpG hypermutability. Thus, the relationship between codon bias and gene expression level can be explained on the basis of the translation-selection model [2]. This model proposes that codon usage in highly expressed genes is biased toward "optimal" codons, i.e., codons corresponding to more abundant tRNAs. This bias has been demonstrated to affect both elongation rate and accuracy [50,51]. As shown in Table 2, the calculated negative correlation indicates that the codons used in this study are not optimal. In human and mouse genomes, the most frequently used codons [52] are not those with the most abundant tRNAs [35].

Recent studies [36,39] have shown that CpG mutation rates in the non-coding regions of the human genome negatively correlate with the local GC content [53-56]. Isochores of the human genome [57] appear to be an influential factor that affects codon composition [53-56], and several studies have shown that this factor is related to gene expression levels [58,59]. However, additional studies are necessary to confirm the relationship between codon bias and the positional effect of genes.

Plotkin *et al. *[24] showed that codon usage for tissue-specific genes varies among the tissues in which such genes are expressed, thereby suggesting that this variation may be affected by differential tRNA-gene copy numbers in different tissues. However, this variability in codon usage among tissues is still under debate [22,60,61]. Nevertheless, it is noteworthy that codon substitutions are affected by adjacent codons [36,39], and are therefore indirectly affected by adjacent amino acids [38]. Amino acid frequencies may also be tissue specific, although additional studies are necessary to investigate the effect of CpG hypermutability on tissue-specific codon usage. Furthermore, codon bias in mammalian genomes should also be investigated with regard to the presence of CpG nucleotides [27,28,30].

## Conclusion

In mouse, the effect of gene expression level on codon bias is weaker than both the effect of gene expression level on amino acid composition and the effect of CpG hypermutability on codon bias. However, to detect the effect of gene expression level on codon bias in mouse, a study of more genes is necessary.

## Competing interests

The authors declare that they have no competing interests.

## Authors' contributions

KM wrote the software and the manuscript. RFK supervised the project. Both authors read and approved the final manuscript.
